# Study Protocol of the Exercise Study: Unraveling Limitations for Physical Activity in Children With Chronic Diseases in Order to Target Them With Tailored Interventions—A Randomized Cross Over Trial

**DOI:** 10.3389/fped.2021.791701

**Published:** 2022-01-13

**Authors:** Linda E. Scheffers, Willem A. Helbing, Elisabeth M. W. J. Utens, Gwen C. Dieleman, Karolijn Dulfer, Josefien Noske, Eline A. van den Broek, Sylvia Walet, Joanne F. Olieman, Johanna C. Escher, Marielle W. Pijnenburg, Ans T. van der Ploeg, Linda E. van den Berg

**Affiliations:** ^1^Department of Pediatric Gastroenterology, Erasmus MC—Sophia Children's Hospital, Rotterdam, Netherlands; ^2^Respiratory Medicine and Allergology, Department of Pediatrics, University Medical Center, Erasmus MC—Sophia Children's Hospital, Rotterdam, Netherlands; ^3^Center for Lysosomal and Metabolic Diseases, Erasmus MC University Medical Center, Rotterdam, Netherlands; ^4^Department of Pediatric Cardiology, Erasmus MC—Sophia Children's Hospital, Rotterdam, Netherlands; ^5^Division of Pediatric Cardiology, Department of Pediatrics, Radboud University Medical Center, Amalia Children's Hospital, Nijmegen, Netherlands; ^6^Department of Child and Adolescent Psychiatry/Psychology, Erasmus MC—Sophia Children's Hospital, Rotterdam, Netherlands; ^7^Research Institute of Child Development and Education, University of Amsterdam, Amsterdam, Netherlands; ^8^Department of Child and Adolescent Psychiatry, Amsterdam University Medical Center/Levvel, Amsterdam, Netherlands; ^9^Intensive Care Unit, Department of Paediatrics and Paediatric Surgery, Erasmus Medical Centre-Sophia Children's Hospital, Rotterdam, Netherlands; ^10^Division of Dietetics, Department of Internal Medicine, Erasmus MC, Rotterdam, Netherlands

**Keywords:** physical exercise, Broncho Pulmonary Dysplasia, inflammatory bowel disease, Fontan—total cavopulmonary, lifestyle intervention, Pompe disease, physical activity, pediatrics

## Abstract

**Introduction:** Physical activity is associated with many physiological and psychological health benefits across the lifespan. Children with a chronic disease often have lower levels of daily physical activity, and a decreased exercise capacity compared to healthy peers. In order to learn more about limitations for physical activity, we investigate children with four different chronic diseases: children with a Fontan circulation, children with Broncho Pulmonary Dysplasia (BPD), Pompe disease and inflammatory bowel disease (IBD). Each of these diseases is likely to interfere with physical activity in a different way. Knowing the specific limitations for physical activity would make it possible to target these, and increase physical activity by a personalized intervention. The aim of this study is to first investigate limitations for physical activity in children with various chronic diseases. Secondly, to measure the effects of a tailored exercise intervention, possibly including a personalized dietary advice and/or psychological counseling, on exercise capacity, endurance, quality of life, fatigue, fear for exercise, safety, muscle strength, physical activity levels, energy balance, and body composition.

**Methods and Analysis:** This randomized crossover trial will aim to include 72 children, aged 6–18 years, with one of the following diagnosis: a Fontan circulation, BPD, Pompe disease and IBD. Eligible patients will participate in the 12-week tailored exercise intervention and are either randomized to start with a control period or start with the intervention. The tailored 12-week exercise interventions, possibly including a personalized dietary advice and/or psychological counseling, will be designed based on the found limitations for physical activity in each disease group during baseline measurements by the Rotterdam Exercise Team. Effects of the tailored training interventions will be measured on the following endpoints: exercise capacity (measured by cardiopulmonary exercise test), endurance, physical activity levels, muscle strength, quality of life, fatigue, fear for exercise, disease activity, cardiac function (in children with a Fontan circulation), energy balance, and body composition.

**Ethics and Dissemination:** Conducted according to the Declaration of Helsinki and Good Clinical Practice. Medical-ethical approval was obtained.

**Trial Registration Number:** NL8181, https://www.trialregister.nl/trial/8181.

## Introduction

Physical activity is of vital importance for the healthy development of children (1, 2). It is associated with many physiological and psychological health benefits across the lifespan. Many children with a chronic disease have lower levels of daily physical activity and lower exercise capacity compared to healthy peers (1). Children with a chronic disease may face obstacles that negatively impact physical activity or sports participation (3, 4). Some conditions may directly influence exercise capacity causing a circulatory, ventilator or peripheral limitation, where others may influence physical activity indirectly due to disease related symptoms like fatigue, dyspnea, diarrhea etc. Even more, sometimes parents are scared to let their sick child participate in sports activities (3). In order to unravel the influence of these burdens on physical activity, we investigate children with four different chronic diseases: children with a Fontan circulation, children with Broncho Pulmonary Dysplasia (BPD), Pompe disease and inflammatory bowel disease (IBD), which all are likely to interfere with physical activity in a different way. Knowing the specific limitations for physical activity might make it possible to target these limitations, and increase physical activity by a tailored intervention. Conceivably, leading to a positive impact on both physiological and psychological health in these children.

Cardiovascular diseases may directly impact exercise capacity causing a circulatory limitation. In children, congenital heart defects are the most frequent type of cardiovascular disease (5). Children with a severe congenital heart defect (like two of the most common: Tetralogy of Fallot or an univentriculair heart) often have impaired exercise capacity and are less physically active compared to healthy peers (5, 6). Several studies have investigated effects of aerobic exercise training in these children, in order to increase exercise capacity and quality of life (7). Duppen et al. showed beneficial effects of aerobic exercise training in children with a Tetralogy of Fallot, but not in children with an univentricular heart threated with a Fontan procedure (6). Strength training, focusing on lower limbs, has been suggested to be more effective in patients with a Fontan circulation due to their different physiology compared to those with a normal (biventricular) heart (8). Despite a very small study in adult patients, this kind of training has never been researched before.

Pulmonary diseases are likely to influence exercise capacity by ventilatory limitation (9). BPD is a common respiratory complication of preterm born children and associated with long-term respiratory problems, including a decreased lung function and decreased exercise capacity (9). Adult survivors with BPD have lower sports participation and physical activity levels compared to healthy peers (10). Although physical exercise training has been proven very effective in various other chronic lung diseases as cystic fibrosis in children and COPD in adults, only one study exists investigating effects of a 4-week exercise program in patients with BPD (11, 12). After 4 weeks lung function improved in these children. However the mechanism of impact on exercise capacity remains unclear, since exercise capacity was not measured in this small study.

Musculoskeletal disorders might cause a peripheral limitation directly influencing exercise capacity. Pompe disease is a lysosomal storage disease caused by a deficiency of the enzyme acid-α-glucosidase, causing a progressive generalized myopathy (13). Treatment with enzyme replacement therapy (ERT) has shown to improve motor function and survival. Despite ERT treatment, many Pompe disease patients eventually become ventilator or wheelchair dependent. It is known that patients with Pompe disease has a decreased exercise capacity which is not caused by impaired skeletal muscle glycogenetic capacity but rather by muscle weakness and wasting (14). A study by van den Berg et al. published in 2015 showed that exercise training in adult patients with late onset Pompe disease might be a safe and effective adjuvant therapy for ERT (15). Research investigating physical training in pediatric Pompe disease patients, and specifically the more severe form Classic Infantile Pompe disease, has not been done before.

Inflammatory bowel disease (IBD), including both ulcerative colitis (UC) and Crohn's disease (CD), is a chronic inflammatory diseases of the gastrointestinal tract, and is one of the most common auto-immune diseases in children (16). IBD is characterized by periods of remission punctuated with relapse. Current standard of care for pediatric IBD patients includes a combination of pharmacotherapy, nutritional support or treatment, and in some cases surgical intervention (17). Despite treatment options, quality of life often remains reduced in children with IBD compared to healthy peers (18). Also, previous studies showed that patients with IBD often have reduced levels of physical activity and a lower exercise capacity compared to peers (19). IBD patients often experience barriers to exercise due to IBD related symptoms such as fatigue, diarrhea and even fear of symptom exacerbation. Evidence supporting that exercise can improve the course of various auto-immune diseases in adult patients is growing (20). An explanation is that physical activity promotes the release of IL-6 from muscles, a myokine possibly causing ant-inflammatory responses. Small studies in adult IBD patients have shown positive effects of exercise with reports of increased exercise capacity, improved fatigue and quality of life and even positive effects on the clinical course of disease (21). Studies investigating the effects of physical activity on inflammation, physical and psychological health in children with IBD and other auto-immune diseases are very limited.

The aim of the exercise study is first to investigate whether children with these chronic diseases are limited in their physical functioning and to unravel the disease specific burden to physical activity. Secondly, to measure the effects of a tailored exercise intervention (possibly including dietary advice and/or psychological counseling) on exercise capacity, endurance, quality of life, fatigue, fear for exercise, safety, muscle strength, physical activity levels, energy balance, and body composition.

## Methods and Analysis

### Study Design

**Figure 1:**
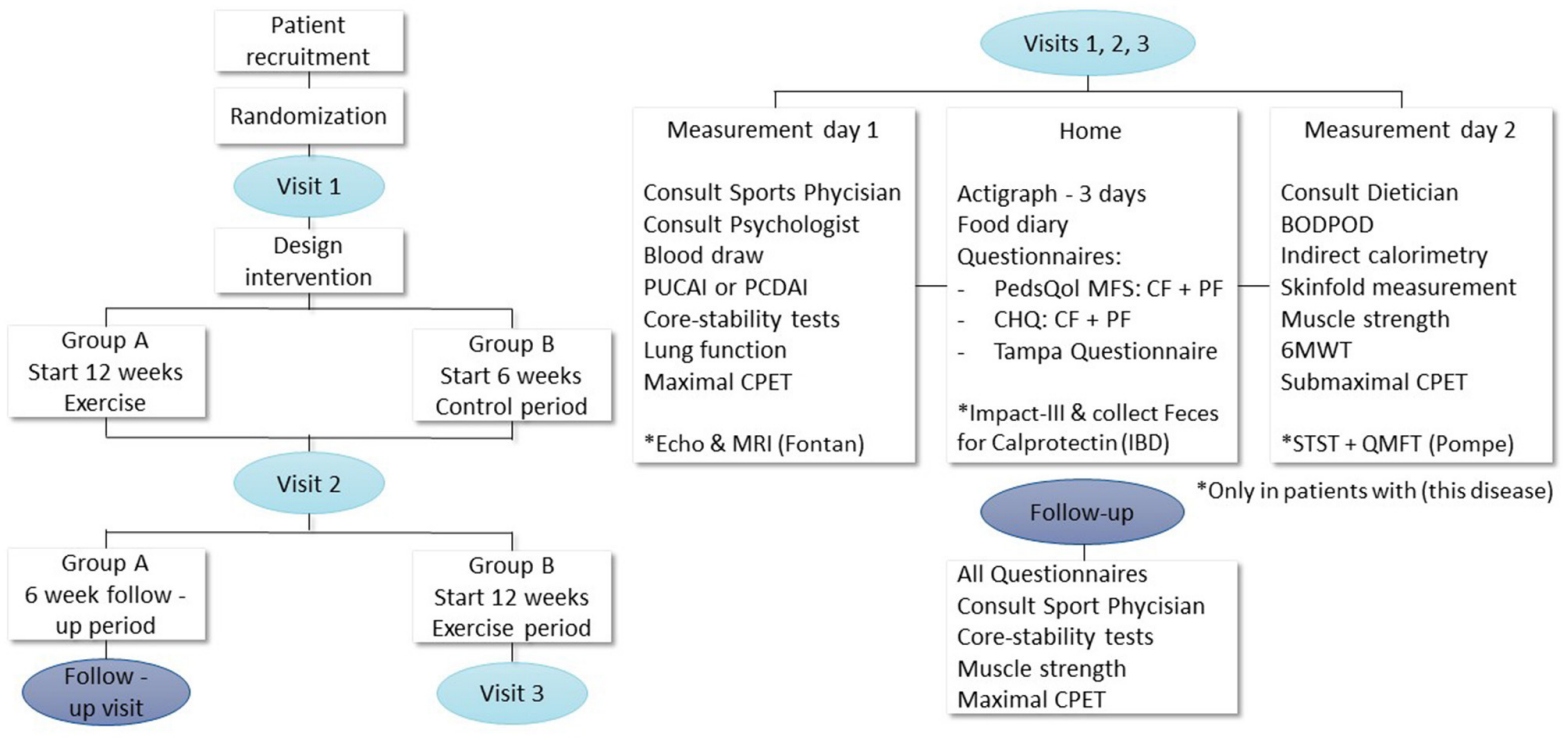
Study design, visits, and measurements. PCDAI, Pediatric Crohn's Disease Activity Index; PUCAI, Pediatric Ulcerative Colitis Activity Index; CPET, cardiopulmonary exercise testing; PedsQol, pediatric quality of life questionnaire; MFS, multifatigue scale; CR and PR, child and parent reports; CHQ, child health questionnaire; 6MWT, 6-minute walking test; BODPOD, body composition measurement; MRI, magnetic resonance imaging; IBD, inflammatory bowel disease; STST, sit to stand test; QMFT, quality motor function test.

This study is a prospective single-center randomized cross-over controlled trial. Eligible patients will participate in the 12-week tailored exercise intervention and are either randomized to start with a control period or start with the intervention. The tailored 12-week exercise interventions will be designed based on the found limitations for physical activity in each disease group during baseline measurements and previously published studies. Effects of the tailored exercise interventions will be measured on the following endpoints: exercise capacity (measured by cardiopulmonary exercise test), endurance, physical activity levels, muscle strength, quality of life, fatigue, fear for exercise, disease activity, cardiac function (in children with a Fontan circulation), energy balance, and body composition. Figure 1 shows the study design, visits, and measurements. The study includes two identical visits and a follow-up moment for group A and three identical visits for group B. Each visit consists of two assessment days with at least 3 days and maximal 7 days in between. All measurements will take place at the Erasmus MC—Sophia Children's hospital, the Netherlands. The study will be performed in accordance with the Declaration of Helsinki. It was approved by the local Ethics Committee of Erasmus MC Medical Centre (NL.70912.078.19) and registered at www.trialregister.nl as Trial NL8181.

### Study Population

In total 72 children will be included in this trial. Children were eligible for inclusion when aged 6–18 years old and diagnosed with one of the following four diseases:

1. Fontan circulation: Patients with a proven univentricular heart using echocardiogram who underwent the Fontan procedure.
2. BPD: Children had to have (1) persistent oxygen demand and/or by means of an oxygen reduction test and (2) abnormalities (hyperinflation, fine reticular lung image and/or atelectasis) on the X-ray of the lungs in a newborn in what would have been week 36 of pregnancy.
3. Pompe disease: Confirmed deficiency of α-glucosidase in leucocytes or lymphocytes, fibroblasts or muscle and/or two pathogenic GAA variants in trans (www.pompevariantdatabase.nl) (22).
4. IBD: Confirmed by ileocoloscopy and esophagogastroduodenoscopy with histology on multiple mucosal biopsies.

Exclusion criteria were:

1. Children <120 cm, as the cardiopulmonary exercise test cannot be executed.
2. Physical inability to perform a cardiopulmonary exercise test (CPET).
3. Participation in organized exercise training programs in a research setting.
4. Medical contra-indications for exercise.

### Randomization

Randomization will be performed in blocks of 4,6 using Castor. All children will be randomized in group A or group B. Group A will start with the tailored intervention after a control period of 6 weeks. Group B will start the tailored intervention immediately after the first measurement moment. Group A serves as controls for the whole group (with a 2:1 ratio) as group A does not have a washout period. Blinding of participants and physiotherapists to allocation is not possible due to the nature of the intervention.

### Sample Size Calculation

The power calculation is based on the primary study outcome of change in peak VO_2_ after the tailored intervention. As reported peak VO_2_ in literature is different for every patient group, the group size needed to observe an increase in peak VO_2_ of at least 5%, which is clinically relevant, is different for each diagnose.

Fontan group size calculation: Previous studies show that children with a single-chamber heart have a Peak VO_2_ of 27 ml/kg/min (23). The difference of a 5% increase in Peak VO_2_ can be observed with a power of 80% and an alpha of 0.05 in 21 children with a unicameral heart assuming a standard deviation of 2.19 VO_2_/kg (24).

*BPD Group Size Calculation*: Average children with BPD have a VO_2max_ of 39 ml/kg/min (25). An improvement in VO_2max_ of at least 5% is considered clinically relevant. The difference of an increase in VO_2max_ of 5% can be observed with a power of 80% and an alpha of 0.05 in a sample of 10 children with BPD, assuming a standard deviation of 2.19 VO_2_/kg (24).

*Pompe Group Size Calculation*: The only previous study investigating exercise capacity in children with Pompe disease includes five patients and shows that children with Pompe have a PeakVO_2_ between 11.1 and 40.2 ml/kg/min, with a 75%ile of 37.7 ml/kg/min (14). We have chosen to use the 75%ile to calculate the group size, as we think this value of PeakVO_2_ is closest to the PeakVO_2_ of our own Pompe children population. An improvement in PeakVO_2_ of at least 5% is considered clinically relevant. The difference of a 5% increase in PeakVO_2_ can be observed with a power of 80% and an alpha of 0.05 at a number of 10 children with Pompe disease assuming a standard deviation of 2.19 VO_2_/kg (24).

*IBD Group Size Calculation*: In previous studies, untrained children with IBD had an average peak VO_2_ of 36 ml/kg/min (19). Twelve IBD patients will be needed in order to observe an increase in peak VO_2_ of at least 5%, which is clinically relevant, with a power of 80% and an alpha of 0.05 based on a standard deviation of 2.19 VO_2_/kg (24).

Anticipated on a dropout rate of 30–40% at 16 patients with IBD, 14 patients with Pompe disease, 28 patients with a Fontan circulation and 14 patient with PBD will be included.

### Intervention

**Figure 2:**
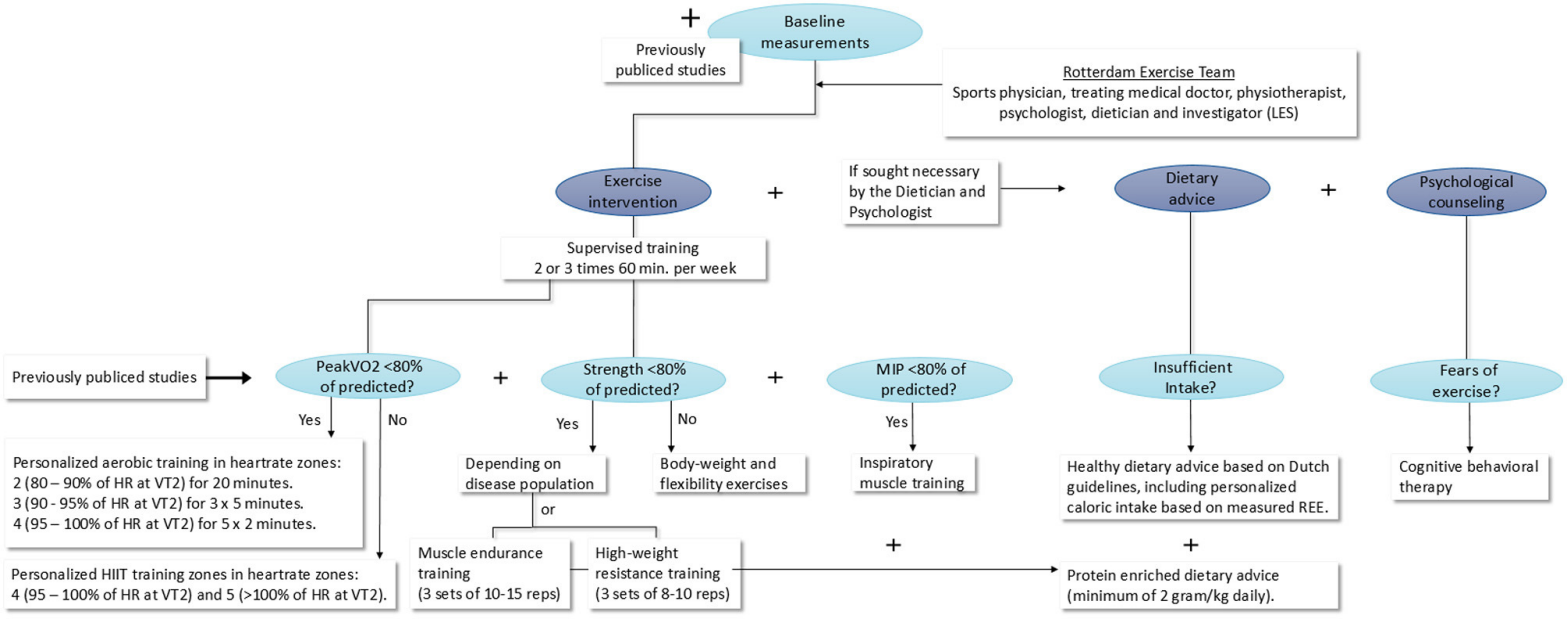
Design process and content of the tailored exercise intervention. MIP, Maximal inspiratory pressure; min, minutes; HIIT, high intensity interval training; HR, heart rate; VT2, ventilatory anaerobic threshold; REE, rest energy expenditure; kg, kilograms.

The tailored 12-week exercise interventions, possibly including a personalized diet advice or/and psychological counseling, will be designed based on the results of the baseline measurements for each disease group. Figure 2 shows this process and the content of the tailored exercise intervention. In order to design the tailored intervention a multidisciplinary team (the Rotterdam Exercise Team), consisting of a sports physician, treating medical doctor, physiotherapist, psychologist, dietician and the investigator (LES) will compare baseline outcomes for each disease group to normal values of healthy peers in order to find possible limitations to physical activity. Based on these findings, the most fitting tailored intervention will be designed by the Rotterdam Exercise Team.

All children will participate in two or three supervised training sessions a week for 12 consecutive weeks. The most fitting form of physical exercise will be determined based on the consult with the sports physician in combination with the outcomes of the Maximal CPET, muscle strength measurements and core-stability measurements and measured maximal inspiratory pressure. Naturally, all previously published studies regarding exercise in the specific disease group will be taken in consideration when designing the tailored exercise intervention. When, for example, a certain training type has already been proven to be ineffective, this type of training will not be added to our tailored exercise intervention.

If a patient group shows a decreased PeakVO_2_ of <80% of predicted during baseline measurements, personalized aerobic training will be added to the exercise intervention consisting of 3 training sessions a week in heartrate zones: 2 (80–90% of HR at VT2) for 20 mins, 3 (90–95% of HR at VT2) for 3 × 5 mins and 4 (95–100% of HR at VT2) for 5 × 2 mins. If a patient group does not show a decreased PeakVO_2_ of <80% of predicted, personalized HIIT in heartrate zones 4 (95–100% of HR at VT2) and 5 (>100% of HR at VT2) will be added to the tailored intervention. Depending on measured muscle strength of the patient groups during the baseline measurements, muscle endurance exercises, high weight resistance exercises or body-weight and flexibility exercises, will be added to the program.

When a patient group shows decreased MIP (<80% of predicted), inspiratory muscle training (IMT) will be added using a Philips threshold IMT device following the previously published clinical guidelines of Hill et al. (26). Training will be given under supervision of a physiotherapist at a practice close to the children's homes. A researcher from the Erasmus MC—Sophia Children's Hospital will supervise all first training sessions and visit a training session of each patient every other week to monitor uniform execution of the tailored interventions. In addition, patients will be telephoned weekly to evaluate training progress (and if added, the dietary advice and psychological counseling) and assure compliance.

In addition to the physical training a personalized dietary advice and/or psychological counseling can be added to the exercise intervention if sought necessary by the Rotterdam Exercise Team.

A personalized dietary advice will be added if a patient group has insufficient intake, this will be determined based on the consult with the dietician, bodyweight and body composition measurements, the REE and the filled in food diaries. The personalized dietary advice will consist out of a healthy eating advice based on Dutch guidelines, including an advised calorie intake based on REE (27). A protein enriched diet of 2 g/kg daily will be added to the dietary advice when muscle endurance exercises or high-weight resistance exercises are included in the tailored exercise intervention.

Psychological counseling will be added to the personalized intervention if sought necessary by the psychologist, for example when fears regarding exercise are present in a disease group, cognitive behavioral therapy will be added. This will be determined based on the psychological consults with parents and children, the quality of life questionnaires, Tampa scale and the fatigue questionnaires.

### Outcome Measurements

#### Primary Study Outcome

1. Exercise capacity: Change in peak oxygen uptake (peak VO_2_), will be measured by maximal cardiopulmonary exercise testing (CPET).

#### Secondary Outcomes

1. Aerobic Fitness
  - Maximal CPET: Oxygen exchange (VO_2_), oxygen consumption per heartbeat (O_2_pulse), ventilatory exchange (VE), load (W), heartrate (HR) and respiratory exchange rate (RER) determined at the ventilatory aerobic threshold (VAT) and at exhaustion (peak).
  - Submaximal CPET: Average heartrate (HRavg) and maximum heartrate (HRmax).
  - Six-Minute Walk Test (6MWT): Maximum walked distance in meters.
2. Physical activity levels: Time in sedentary state and time in moderate activity will be measured using the ActiGraph GT3X accelerometer and structured interview.
3. Health related quality of life: Will be measured using Child Health Questionnaires (CHQ-PF28 & CHQ-CF45).
4. Fatigue: Will be measured by the Multidimensional Fatigue Scale questionnaire (PedsQolMFS).
5. Fear of physical exercise: Will be measured using the TAMPA scale for kinesiophobia questionnaire and the kinesiophobia meter during the psychological consult.
6. Muscle strength: Elbow flexion and extension, shoulder abduction, hip flexion and abduction, knee extension and flexion and squeezing strength will be measured using Hand-Held-Dynamometry (HHD).
7. Core stability: Will be measured by time in seconds in balance for four core stability tests.
8. Energy balance: Caloric intake estimated using a food diary, resting energy expenditure (REE) measured with indirect calorimetry.
9. Body composition: BMI, body weight and body fat percentage, will be measured using both BOD POD and four side skinfold method.
10. Lung function: Forced vital capacity (FVC), forced expiratory volume (FEV1) and FVC/FEV1 ratio will be measured using a spirometry tests.
11. Effects on the following laboratory outcomes: IGF-1, CK, Growth hormone.

#### Disease Specific Secondary Outcomes

1. For children with a Fontan circulation: An echocardiogram, magnetic resonance imaging and NT-pro-BNP measurement will be added to assess cardiac function.
2. For children with BPD: Lung function will be measured before and after the maximal CPET.
3. For Pompe patients: Muscle functioning using the Quick Motor Function Test (QMFT) and Supine To Stand Test (STST).
4. For IBD patients: Effects on disease activity using the PCDAI and PUCAI scores and calprotectin will be measured in Feces. In addition to the CHQ, children will also fill in the Impact-III questionnaire to asses quality of life.

### Aerobic Fitness

Endurance will be assessed by maximal CPET, submaximal CPET, and 6-minute walking test (6MWT). All patients will undergo the maximal CPET, and submaximal CPET on the same electrically braked bicycle ergometer: Jaeger ER9000 (Viasys Healthcare, Hoechberg, Germany). The maximal CPET starts with 1 min of unloaded cycling with a rate of 60–80 repetitions per minute where after exercise intensity will be increased progressively until exhaustion (ramp protocol), the test ends with a 3 min recovery phase. The rate of increase will be considering the patient's functional capacities.

The submaximal CPET starts with 3 mins of unloaded cycling, followed by 6 mins of cycling with an exercise intensity set on half of the previously reached maximum workload during the maximal CPET, and ends with a 3-minute recovery phase. During both maximal and submaximal CPET, respiratory parameters and heart rhythm will continuously be measured using respectively breath by breath analyses: Oxycon Champion System (Viasys Healthcare, Conshohocken, United States) and 12 lead ECG. Blood pressure will be measured every 2 mins. Exercise tests with a peak respiratory exchange rate (RER) ≥ 1.00, a Heartrate >85% of calculated maximal heart rate and lung capacity <20% during the test will be considered maximal. The following parameters will be determined from the maximal CPET: Peak VO_2_ in ml/kg, peak minute ventilation (VEpeak), peak workload (Wpeak), peak heart rate (HRpeak), peak oxygen pulse (peakO_2_Pulse), peak RER, and ventilatory anaerobic threshold (VAT). The VAT will be assessed independently by two researchers using the ventilatory equivalents method. The following parameters will be determined from the submaximal CPET: average heart rate and HRpeak.

The 6MWT will be performed in accordance with the American Thoracic Society guidelines, however due to a lack of space the course will be 8 m of length instead of 30 m.

### Physical Activity Levels

During the consult with the Sports Physician, children and parents will be asked about the amount of time spend on physical activity a week. Subsequently, physical activity levels will be measured with a validated Actigraph GT3X+ accelerometer (firmware v3.2.1, ActiGraph Inc, Pensacola, FL, USA). The subjects will wear the accelerometer on their right hip for 2 week days and 1 weekend day. The actigraph will be removed only for swimming, showering, and sleeping. The Actigraph was set into tri-axial mode, using a 10-s epoch and 30 Hz. Non-wear time will be assessed using the Troiano protocol (28). Data will be analyzed with ActiLife® software (v6.10.2), counts per minute will be converted to metabolic equivalent (MET) using age specific formulas by Freedson et al. (29).

### Quality of Life, Fatigue, and Fear of Exercise

A semi-structured interview will be completed by a psychologist for both parents and children separately. In these interviews, biographical data, such as household composition, educational level, social participation, social-economic status, fear of exercise (answered by parents and children), perceived body image and quality of life (answered by parents and children) will be assessed.

The child health questionnaire (CHQ) child form (CF) and Parent form (PF) and the IMPACT-III questionnaire (for IBD patients) will be filled in at home and used to assess health-related quality of life (HRQol) (30). The PedsQL Multidimensional Fatigue Scale (MFS) CF and PF will be used to evaluate the effect of the exercise training on Fatigue (31). The TAMPA questionnaire and kinesiophobia questionnaire assess potential kinesiophobia (32).

### Core Stability and Muscle Strength

All muscle strength measurements will be performed in a standardized manner by the same trained investigator (LES) using Hand-held Dynamometry (HHD). To assess core stability LES measures time in balance for each of the following four core stability exercises: plank, back bridge, left side bridge, and right side bridge.

### Body Composition and Energy Balance

At each measurement moment patients the height, weight, and body composition, using a skinfold caliper (four skinfolds method) and air displacement plethysmography (ADP) on whole-body densitometry using the BOD POD (BOD POD body composition system, COSMED, Ltd, Concord, CA, USA) will be measured. Percent body fat (%BF) will be calculated using the equations published by Brook, Drunin, Rahaman and Wormsley et al. for the skinfold measurements, and the Lohman equation for the ADP (33–35).

All patients will keep up with a detailed food diary for three consecutive days and undergo an indirect calorimetry (Life Measurements, Inc, Concord, CA, USA), during the consult with the dietician to measure rest expenditure (REE). Measurements are performed after a fasting period of at least 2 h. Both measured REE and estimated REE, calculated using the Scholfield equation, are then converted to Total energy expenditure (TEE) (36–38).

### Lung Function

Forced vital capacity (FVC), forced expiratory volume (FEV1) and FVC/FEV1 ratio will be measured using an electronic spirometer (Masterscreen PFT; Carefusion; San Diego, CA) by a trained researcher.

### Laboratory Outcomes

At each measurement day blood samples will be collected from a peripheral vein and analyzed in the clinical laboratory of the Erasmus MC—Sophia Children's hospital, the following markers will be assessed in all patient groups: IGF-1, Creatine Kinase (CK), Growth hormone (GH). In addition, erythrocyte sedimentation rate (ESR), C-reactive protein (CRP) and albumin will also be assessed in IBD patients. In children with a Fontan circulation, N-terminal pro-brain natriuretic peptide (NT-proBNP) will be measured.

### IBD Specific Measurements

To assess the effects of the training program on disease activity: fecal calprotectin, lab measurements and PCDAI or PUCAI will be observed at each measurement moment. Remission is defined as PCDAI < 10 or PUCAI < 10, mild disease activity as PCDAI 10–27.5 and PUCAI 10–34, moderate disease activity as PCDAI 30–37.5 and PUCAI 35–64, severe disease activity is defined as PCDAI ≥ 40 and PUCAI ≥ 65 (39, 40).

### Pompe Disease Specific Measurements

In Pompe patients muscle function will be assessed using the Quick Motor Function Test (QMFT). The QMFT is a tool specifically constructed to measure motor function of Pompe disease patients (41). The test contains 16 motor function items that are specifically difficult for patients with Pompe disease and reflect daily life activities. Each item can be scored 0 (unable to perform) to 4 (can easily perform). Scores of each item are summed. The maximum score of 64 indicates normal muscle functioning. Additionally, the supine to stand test (STST) will be taken in order to assess the ability of a patient to rise from the floor. The STST measures the required time in seconds for a patient to move from a supine to standing position.

### Fontan Specific Measurements

Patients will undergo transthoracic echocardiography and cardiac magnetic resonance before and after the tailored interventions. Echocardiograms will be performed by experienced echocardiographic technicians, following the recommendations of the American Society of Echocardiography (42). All studies will be obtained in rest using the using the Philips EPIC7C (Andover, MA, USA) and Vivid E9 (General Electric Vingmed Ultrasound, Horten, Norway) ultrasound systems. Images will be collected with an appropriate transducer, based on age and weight of the participant. Images will be recorded and analyzed of-line using Intellispace Cardiovascular software (Philips, Best, The Netherlands). We measure the following cardiac parameters: maximum velocity at the aorta valve, aorta descendens and across the atrioventricular (AV) valve of the dominant ventricle, as well as deceleration time of peak velocity across the AV valve, using optimal Doppler settings. M-mode will be used to assess maximal annular plane systolic excursions (APSE) at the lateral side of the dominant AV-valve.

Cardiac magnetic resonance imaging (MRI) will be performed on whole body MRI scanners (GE Signa MR/I 1.5 T, USA; GE Discovery MR 450, USA). A multi-phase, multi-slice volumetric data set will be acquired using a fast 2D cine scan using steady-state free precession (SSFP). Trough plane 2D phase contrast MRI will be used for flow measurements. All images will be acquired with either breath-holds or over 3 heart beats to eliminate respiratory effects. Analysis will be performed with the software packages QMASS and QFLOW (Medis Medical Imaging Systems, The Netherlands). The following cardiac parameters will be assessed: end-diastolic volume (EDV), end-systolic volume (ESV), stroke volume (SV), ejection fraction (EF), ventricular mass. The following flow measurements will be performed: above aoortic valve, on aortic valve, aorta on diaphragm level, vena cava inferior (VCI), vena cava superior (VCS), arteria pulmonalis dextra (APD), arteria pulmonalis sinistra (APS) and mitral valve and/or tricuspid valve flow. All assessments will be executed with manual contour detection. If a second ventricle exists, the volume will be assessed and added to the systemic ventricle and presented as a single ventricle. All MRI results will be obtained from analysis by an experienced pediatric cardiologist (WH).

### Statistical Analysis

Data will be collected in the Castor online database. Patient characteristics will be described using descriptive statistics and tested for normality (using both the shapiro-wilk test and a visual evaluation of the histograms). A per protocol analysis will be used. Baseline characteristics between groups will be compared with the unpaired student's T-test for normally distributed data, Mann-Whitney U for not normally distributed data and Chi-squared test for proportions. Differences before and after the control or exercise period will be analyzed using a paired T-test for normally distributed data and Wilcoxon signed ranks test for not normally distributed data. If possible, missing outcome data will be imputed using multiple imputation. Statistical analyses will be performed using SPSS or R software. The significance level is determined at *P* < 0.05.

## Discussion

The Exercise study is a unique study investigating limitations for physical activity in children with a four chronic diseases: children with a Fontan circulation, children with Broncho Pulmonary Dysplasia (BPD), Pompe disease and inflammatory bowel disease (IBD). Each of these diseases is likely to interfere with physical activity in a different way. Knowing the specific limitations for physical activity would make it possible to target these, and increase physical activity by a personalized intervention. Clinical intervention trials investigating safety and potential beneficial effects of tailored exercise interventions in children, are very limited. In the Exercise study a tailored intervention program, possibly including a personalized diet advice or/and psychological counseling, for each patient group will be designed by a multidisciplinary team, based on the baseline findings. The effects of these tailored interventions will be measured on both patient-related outcomes and important disease-related outcomes. This study thereby first aims to obtain more information about limitations for physical activity in children with various chronic diseases. And secondly, investigates the safety and potential benefits of exercise in chronic ill children, so better tailored and disease specific advice surrounding exercise can be given by doctors to their patients in the future.

## Author Contributions

MP, EU, GD, KD, JO, AP, WH, JE, SW, LB, and LS designed the protocol together. LS drafted this manuscript. MP, EU, GD, KD, JO, AP, WH, JE, SW, and LB critically revised the manuscript and approved the final version. All authors helped writing the protocol that was approved by the medical ethical commission.

## Funding

Vrienden van Sophia granted the funds for the Exercise study.

## Conflict of Interest

The authors declare that the research was conducted in the absence of any commercial or financial relationships that could be construed as a potential conflict of interest.

## Publisher's Note

All claims expressed in this article are solely those of the authors and do not necessarily represent those of their affiliated organizations, or those of the publisher, the editors and the reviewers. Any product that may be evaluated in this article, or claim that may be made by its manufacturer, is not guaranteed or endorsed by the publisher.

## References

[1] West SL Banks L Schneiderman JE Caterini JE Stephens S White G . Physical activity for children with chronic disease; a narrative review and practical applications. BMC Pediatr. (2019) 19:12. 10.1186/s12887-018-1377-3 30621667 PMC6325687

[2] Harkel ADJT Takken T. Exercise testing and prescription in patients with congenital heart disease. Int J Pediat. (2010) 2010:791980. 10.1155/2010/791980 20871857 PMC2943096

[3] Shields N Synnot A. Perceived barriers and facilitators to participation in physical activity for children with disability: a qualitative study. BMC Pediatr. (2016) 16:9. 10.1186/s12887-016-0544-7 26786677 PMC4717582

[4] Bar-Or O. Pathophysiological factors which limit the exercise capacity of the sick child. Med Sci Sports Exerc. (1986) 18:276–82. 10.1249/00005768-198606000-00004 3724407

[5] van der Linde D Konings EE Slager MA Witsenburg M Helbing WA Takkenberg JJ . Birth prevalence of congenital heart disease worldwide: a systematic review and meta-analysis. J Am Coll Cardiol. (2011) 58:2241–7. 10.1016/j.jacc.2011.08.025 22078432

[6] Duppen N Etnel JR Spaans L Takken T. Does Exercise Training Improve Cardiopulmonary Fitness and Daily Physical Activity in Children and Young Adults With Corrected Tetralogy of Fallot or Fontan. Amsterdam: Elsevier (2015) 10.1016/j.ahj.2015.06.018 26385046

[7] Scheffers LE Berg LEMv Ismailova G Dulfer K Takkenberg JJM Helbing WA. Physical exercise training in patients with a Fontan circulation: a systematic review. Eur J Prevent Cardiol. (2020) 2020:2047487320942869. 10.1177/2047487320942869 34551076

[8] Cordina RL O'Meagher S Karmali A Rae CL Liess C Kemp GJ . Resistance training improves cardiac output, exercise capacity and tolerance to positive airway pressure in Fontan physiology. Int J Cardiol. (2013) 168:780–8. 10.1016/j.ijcard.2012.10.012 23154055

[9] Kalikkot Thekkeveedu R Guaman MC Shivanna B. Bronchopulmonary dysplasia: A review of pathogenesis and pathophysiology. Respir Med. (2017) 132:170–7. 10.1016/j.rmed.2017.10.014 29229093 PMC5729938

[10] Gray PH O'Callaghan MJ Poulsen L. Behaviour and quality of life at school age of children who had bronchopulmonary dysplasia. Early Hum Dev. (2008) 84:1–8. 10.1016/j.earlhumdev.2007.01.009 17317043

[11] Morales Mestre N Papaleo A Morales Hidalgo V Caty G Reychler G. Physical activity program improves functional exercise capacity and flexibility in extremely preterm children with bronchopulmonary dysplasia aged 4-6 years: a randomized controlled trial. Arch Bronconeumol. (2018) 54:607–13. 10.1016/j.arbres.2018.05.001 30518495

[12] Burr JF Davidson W Shephard RJ Eves N. Physical activity in chronic respiratory conditions: assessing risks for physical activity clearance and prescription. Can Fam Physician. (2012) 58:761–4. 22859640 PMC3395516

[13] van der Ploeg AT Reuser AJ. Pompe's disease. Lancet. (2008) 372:1342–53. 10.1016/S0140-6736(08)61555-X 18929906

[14] Bar-Yoseph R Mandel H Mainzer G Gur M Tal G Shalloufeh G . Cardiopulmonary exercise test to quantify enzyme replacement response in pediatric Pompe disease. Pediatr Pulmonol. (2018) 53:366–73. 10.1002/ppul.23830 29356433

[15] van den Berg LE Favejee MM Wens SC Kruijshaar ME Praet SF Reuser AJ . Safety and efficacy of exercise training in adults with Pompe disease: evalution of endurance, muscle strength and core stability before and after a 12 week training program. Orphanet J Rare Dis. (2015) 10:87. 10.1186/s13023-015-0303-0 26187632 PMC4506616

[16] Rosen MJ Dhawan A Saeed SA. Inflammatory Bowel Disease in Children and Adolescents. JAMA Pediatr. (2015) 169:1053–60. 10.1001/jamapediatrics.2015.1982 26414706 PMC4702263

[17] Escher JC Taminiau JA Nieuwenhuis EE Büller HA Grand RJ. Treatment of inflammatory bowel disease in childhood: best available evidence. Inflamm Bowel Dis. (2003) 9:34–58. 10.1097/00054725-200301000-00006 12656136

[18] van den Brink G Stapersma L Vlug L Rizopolous D Bodelier A van Wering H . P205 Prevalence and risk factors for anxiety and depressive symptoms in children, adolescents and young adults with inflammatory bowel disease. J Crohn's Colitis. (2018) 12(supplement_1):S202–S3. 10.1093/ecco-jcc/jjx180.332

[19] Ploeger HE Takken T Wilk B Issenman RM Sears R Suri S . Exercise capacity in pediatric patients with inflammatory bowel disease. J Pediatr. (2011) 158:814–9. 10.1016/j.jpeds.2010.10.020 21146188

[20] Sharif K Watad A Bragazzi NL Lichtbroun M Amital H Shoenfeld Y. Physical activity and autoimmune diseases: get moving and manage the disease. Autoimmun Rev. (2018) 17:53–72. 10.1016/j.autrev.2017.11.010 29108826

[21] Engels M Cross RK Long MD. Exercise in patients with inflammatory bowel diseases: current perspectives. Clin Exp Gastroenterol. (2018) 11:1–11. 10.2147/CEG.S120816 29317842 PMC5743119

[22] Center P. www.pompevariantdatabase.nl: Erasmus MC—Sophia Pediatric Hospital (2021). Available online at: www.pompevariantdatabase.nl (accessed December 16, 2021).

[23] Duppen N Etnel JR Spaans L Takken T Van Den Berg-Emons RJ Boersma E . Does exercise training improve cardiopulmonary fitness and daily physical activity in children and young adults with corrected tetralogy of Fallot or Fontan circulation? A randomized controlled trial. Am Heart J. (2015) 170:606–14. 10.1016/j.ahj.2015.06.018 26385046

[24] Barron A Dhutia N Mayet J Hughes AD Francis DP Wensel R. Test-retest repeatability of cardiopulmonary exercise test variables in patients with cardiac or respiratory disease. Eur J Prev Cardiol. (2014) 21:445–53. 10.1177/2047487313518474 24398370

[25] Zavorsky GS Kryder JR Jacob SV Coates AL Davis GM Lands LC. Exercise capacity of children with pediatric lung disease. Clin Invest Med. (2009) 32:E302. 10.25011/cim.v32i6.10666 20003836

[26] Hill K Cecins NM Eastwood PR Jenkins SC. Inspiratory muscle training for patients with chronic obstructive pulmonary disease: a practical guide for clinicians. Arch Phys Med Rehabil. (2010) 91:1466–70. 10.1016/j.apmr.2010.06.010 20801269

[27] Stichting Voedingscentrum Nederland (2021). Available online at: https://www.voedingscentrum.nl/nl.aspx (accessed December 01, 2021).

[28] Troiano RP. Large-scale applications of accelerometers: new frontiers and new questions. Med Sci Sports Exerc. (2007) 39:1501. 10.1097/mss.0b013e318150d42e 17805080

[29] Sasaki JE John D Freedson PS. Validation and comparison of ActiGraph activity monitors. J Sci Med Sport. (2011) 14:411–6. 10.1016/j.jsams.2011.04.003 21616714

[30] Abdovic S Mocic Pavic A Milosevic M Persic M Senecic-Cala I Kolacek S. The IMPACT-III (HR) Questionnaire: a valid measure of health-related quality of life in Croatian children with inflammatory bowel disease. J Crohn's Colitis. (2013) 7:908–15. 10.1016/j.crohns.2012.12.010 23333037

[31] Varni JW Burwinkle TM Szer IS. The PedsQL Multidimensional Fatigue Scale in pediatric rheumatology: reliability and validity. J Rheumatol. (2004) 31:2494–500. 10.3109/17477160903111706 15570657

[32] Weermeijer JD Meulders A. Clinimetrics: Tampa scale for kinesiophobia. J Physiother. (2018) 64:126. 10.1016/j.jphys.2018.01.001 29567379

[33] Brook CG. Determination of body composition of children from skinfold measurements. Arch Dis Child. (1971) 46:182–4. 10.1136/adc.46.246.182 5576028 PMC1647464

[34] Durnin JV Rahaman MM. The assessment of the amount of fat in the human body from measurements of skinfold thickness. Br J Nutr. (1967) 21:681–9. 10.1079/BJN19670070 6052883

[35] Durnin JV Womersley J. Body fat assessed from total body density and its estimation from skinfold thickness: measurements on 481 men and women aged from 16 to 72 years. Br J Nutr. (1974) 32:77–97. 10.1079/BJN19740060 4843734

[36] Schofield WN. Predicting basal metabolic rate, new standards and review of previous work. Hum Nutr Clin Nutr. (1985) 39 Suppl 1:5–41. 4044297

[37] Organization WWH. Energy and Protein Requirements, Report of a Joint FAO/WHO/UNU Expert Consultation. Geneva: World Health Organization. (1985). 3937340

[38] Harris JA. A Biometric Study of Basal Metabolism in Man. Washington, DC: Carnegie Institute of Washington (1919).

[39] Hyams JS Ferry GD Mandel FS Gryboski JD Kibort PM Kirschner BS . Development and validation of a pediatric Crohn's disease activity index. J Pediatr Gastroenterol Nutr. (1991) 12:439–47. 10.1097/00005176-199105000-00005 1678008

[40] Turner D Otley AR Mack D Hyams J de Bruijne J Uusoue K . Development, validation, and evaluation of a pediatric ulcerative colitis activity index: a prospective multicenter study. Gastroenterology. (2007) 133:423–32. 10.1053/j.gastro.2007.05.029 17681163

[41] van Capelle CI van der Beek NAME de Vries JM van Doorn PA Duivenvoorden HJ Leshner RT . The quick motor function test: a new tool to rate clinical severity and motor function in Pompe patients. J Inherit Metab Dis. (2012) 35:317–23. 10.1007/s10545-011-9388-3 21912959 PMC3278629

[42] Lai WW Geva T Shirali GS Frommelt PC Humes RA Brook MM . Guidelines and standards for performance of a pediatric echocardiogram: a report from the Task Force of the Pediatric Council of the American Society of Echocardiography. J Am Soc Echocardiogr. (2006) 19:1413–30. 17138024 10.1016/j.echo.2006.09.001

